# Data on mutations and Clinical features in SCN1A or SCN2A gene

**DOI:** 10.1016/j.dib.2018.08.122

**Published:** 2018-08-30

**Authors:** Yanting Kong, Kai Yan, Liyuan Hu, Mingbang Wang, Xinran Dong, Yulan Lu, Bingbing Wu, Huijun Wang, Lin Yang, Wenhao Zhou

**Affiliations:** a Division of Neonatology, Children׳s Hospital of Fudan University, Shanghai, China; b Division of Endocrinology, Genetics and Metabolic Diseases, Children׳s Hospital of Fudan University, Shanghai, 201102, China; c Key Laboratory of Birth Defects, Children׳s Hospital of Fudan University, Shanghai, China; d Key Laboratory of Neonatal Diseases, Ministry of Health, Children׳s Hospital of Fudan University, Shanghai, China

## Abstract

Mutations in *SCN1A* and *SCN2A* are associated with a wide spectrum of epilepsy related disorders in human. This dataset presented variants and clinical features of SCN1A and SCN2A genes. A total of 48 cases were presented, including 33 *SCN1A* mutations and 14 *SCN2A* mutations. While 22 mutations were novel in *SCN1A* and 11 were novel in *SCN2A*. The clinical features were included of gender, birth history, family history, seizure onset age, seizure types, frequency of seizures, initial and follow-up EEGs, brain MRI findings, antiepileptic drugs, prognosis and developmental data. The data can provide insights on novel mutations and different phenotypes of *SCN1A* and *SCN2A*.

**Specifications table**

**Table:** 

Subject area	*Genetics*
More specific subject area	*Seizures*
Type of data	*Table*
How data was acquired	*Next-generation sequencing, clinical data*
Data format	*Analyzed*
Experimental factors	*Sample was peripheral blood*
Experimental features	*SCN1A and SCN2A variants were identified by next-generation sequencing. Variants which assumed to be pathogenic were identified.*
Data source location	*Shanghai, China*
Data accessibility	*Data are available in this article*

**Value of the data**

• The data identify novel mutations in sodium channel SCN1A and SCN2A genes.
• The data show the difference of EEGs and prognosis between *SCN1A* and *SCN2A* mutations.
• The data might be useful for further function experiments in cellular and animal levels.

## 1 Data

Mutations in genes encoding the voltage-gated sodium channel are associated with seizure-related syndromes. Different genes or different mutations in the same gene have distinct phenotypes [1], [2]. Clinical features and mutations of SCN1A and SCN2A genes are presented in Table 1, Table 2.

**Table 1:** Summary of the clinical features of subjects with a *SCN1A* mutation.

Patient	1	2	3	4	5
Sex	M	F	M	M	F
history of pregnancy and delivery	asphyxia	normal	normal	normal	normal
Family history	NA	(–)	(–)	(–)	(–)
Diagnosis	neonatal seizure	neonatal seizure	Dravet syn	epilepsy	Dravet syn
Mutation	NM_001165963:exon11:c.1978C>A(p.P660T), NA	NM_001165963:exon11:c.1888C>T(p.R630W), paternal	NM_001165963:exon15:c.2825_2826insT,NA	NM_001165963:exon3:c.437 C>G(p.T146R) *de novo*	NM_001165963:exon15:c.2927 T>A(p.M976K) *de novo*
HGMD	N	Y	N	N	N
Age at onset	1 d	24 d	4 m	5 m	3 m
Initial epileptic attacks	NA	tonic sz	tonic-clonic without fever	tonic-clonic without fever	focal seizure
Frequency of initial sz	NA	1/d	1 /m	1/2 m	5/10d
Transition to other sz	NA	no	tonic-clonic with/without fever, SE	tonic-clonic without fever, SE	tonic-clonic with fever, SE
Initial EEG, age	NA	frontal sharp wave and sharp and slow wave complex	normal	normal	normal
EEG follow up	NA	normal(3 m)	normal	left frontal sharp and slow wave complex(2 y)	central sharp and slow wave complex and spike and slow wave complex(5 m)
MRI findings	corpus callosum and cerebellar hypoplasia	normal	Compression of the cerebellum	Full lateral ventricles	normal
drugs	NA	no	VPA+TPM, OXC, LTG	VPA+TPM, LEV	VPA+TPM
Sz prognosis	NA	no sz	seizure reduction	seizure reduction	seizure reduction
Development	NA	normal(1 y)	severe developmental delay(6 y)	normal(3 y)	mild developmental delay(2 y)

Patient	6	7	8	9	10
Sex	M	M	F	F	F
history of pregnancy and delivery	NA	NA	normal	normal	normal
Family history	NA	grand-uncle	(–)	(–)	(–)
Diagnosis	FS+	epilepsy	Dravet syn	FS+	Dravet syn
Mutation	NM_001165963:exon2:c.274 G>C(p.V92L),NA	NM_001165963:exon23:c.4385_4389delACTTT,NA	NM_001165963:exon10:c.1400 C>G(p.S467X), *de novo*	NM_001165963:exon15:c.2839 G>T(p.V947L),*de novo*	NM_001165963:exon26:c.4985_4989delCTTTG, NA
HGMD	N	N	N	N	N
Age at onset	8 m	11 m	3 m	4 m	7 m
Initial epileptic attacks	tonic-clonic with fever,SE	focal seizure	focal seizure	focal seizure	tonic-clonic
Frequency of initial sz	5/6 m	4/4 m	several times/d	1 /m	1 /m
Transition to other sz	tonic-clonic with/without fever	NA	tonic-clonic with/without fever; myoclonus	NA	tonic-clonic,SE
Initial EEG, age	NA	NA	left temporal sharp wave and sharp and slow wave complex	sharp wave and sharp and slow wave complex in the right front	Right temporal occipital sharp wave and spike wave
EEG follow up	normal	NA	normal	NA	NA
MRI findings	normal	NA	normal	normal	normal
drugs	VPA, LEV	VPA	VPA+LEV+TPM, ketogenic diet	VPA+LEV+LTG, TPM	VPA+TPM+LEV
Sz prognosis	seizure free	NA	seizure reduction	seizure reduction	seizure reduction
Development	normal(4 y)	NA	severe developmental delay(21 m)	normal(4 y)	mild developmental delay(22 m)

Patient	11	12	13	14	15

Sex	M	F	M	F	F
history of pregnancy and delivery	normal	normal	asphyxia	normal	NA
Family history	mother, aunt	(–)	(–)	father	NA
Diagnosis	Dravet syn	Dravet syn	Dravet syn	GEFS+	epilepsy
Mutation	NM_001165963:exon14:c.2589+2 T>A, NA	NM_001165963:exon8:c.1150 T>C(p.W384R),NA	NM_001165963:exon14:c.2573 T>C(p.L858P), *de novo*	NM_001165963:exon26:c.5020 G>A(p.G1674S),NA	NM_001165963:exon23:c.4348 C>T(p.Q1450X) *de novo*
HGMD	N	Y	N	Y	N
Age at onset	6 m	3 m	1 m	8 m	<1 y
Initial epileptic attacks	tonic-clonic	focal seizure	focal seizure	tonic-clonic with fever	NA
Frequency of initial sz	3 /m	1/2–3 m	2–3/d	4–5/y	NA
Transition to other sz	tonic-clonic with/without fever;focal seizure	no	tonic-clonic without fever	no	NA
Initial EEG	spike wave and spike and slow wave complex in the left central-occipital	normal	multifocal sharp wave, spike wave and sharp and slow wave complex	parietal-occipital spike wave and sharp wave	NA
EEG follow up	normal(1y9m)	normal(5 m)	multifocal sharp wave, spike wave and sharp and slow wave complex	normal(6 y)	NA
MRI findings	arachnoid cyst	cerebral dysplasia,thin corpus callosum	cerebral atrophy,thin corpus callosum	CT:normal	NA
drugs	VPA+LEV	VPA+LEV	VPA+TPM+LEV, OXC, CZP	OXC, LEV	VPA+TPM
Sz prognosis	seizure reduction	seizure reduction	seizure reduction	seizure free after LEV	NA
Development	mild developmental delay(3 y)	mild developmental delay(2 y)	severe developmental delay(3 y)	normal(9 y)	NA

Patient	16	17	18	19	20

Sex	M	M	M	F	M
history of pregnancy and delivery	asphyxia	normal	NA	normal	normal
Family history	(–)	(–)	(–)	(–)	great-uncle
Diagnosis	Dravet syn	Dravet syn	FS+	FS+	Dravet syn
Mutation	NM_001165963:exon24:c.4529 C>T(p.A1510V), *de novo*	NM_001165963:exon16:c.2948delC, *de novo*	NM_001165963:exon26:c.5347 G>A(p.A1783T), NA	NM_001165963:exon26:c.5183 G>A(p.G1728E) *de novo*	NM_001165963:exon10:c.1662G>A(p.Q554Q), *de novo*
HGMD	N	N	Y	N	Y
Age at onset	3 m	6 m	5 m	7 m	5 m
Initial epileptic attacks	tonic-clonic without fever	focal seizure	tonic-clonic with fever	tonic-clonic without fever	tonic with fever
Frequency of initial sz	1/1 m	NA	2–3 /m	2/d	1–2 /m
Transition to other sz	focal seizure	tonic-clonic with fever	NA	tonic-clonic with fever	tonic-clonic without fever
Initial EEG	multifocal sharp wave and sharp and slow wave complex	normal	occipital spike wave and spike and slow wave complex	normal	NA
EEG follow up	multifocal sharp wave and sharp and slow wave complex(15 m)	cental spike wave	NA	multifocal sharp wave(22 m)	occipital spike wave and spike and slow wave complex
MRI findings	thin corpus callosum	normal	normal	normal	Abnormal signal of left frontal lobe
drugs	LEV+VPA+TPM	OXC, VPA+TPM+LEV, CZP	VPA+LEV	VPA, OXC,LEV	VPA+LEV+TPM, OXC
Sz prognosis	seizure reduction	seizure free	no effect	seizure free	no effect
Development	severe developmental delay(2 y)	severe developmental delay(4 y)	NA	normal(28 m)	severe developmental delay(12 y)

Patient	21	22	23	24	25

Sex	F	M	M	F	M
history of pregnancy and delivery	normal	normal	normal	normal	normal
Family history	(–)	(–)	mother	(–)	(–)
Diagnosis	epilepsy	FS+	Dravet syn	epileptic	epilepsy
Mutation	NM_001165963:exon13:c.2351_2351delinsCTTAAATACTCTTTT, *de novo*	NM_001165963:exon21:c.4083 C>A(p.F1361L), *de novo*	NM_001165963:exon15:c.2831_2832delGT, *de novo*	NM_001165963:exon11:c.2039 A>G(p.D680G),NA	NM_001165963:exon1:c.82 C>T(p.R28C),NA
HGMD	N	N	N	N	Y
Age at onset	6 m	10 m	3 m	5 y	2 y
Initial epileptic attacks	focal seizure	tonic-clonic with fever	clonic with fever, SE	focal seizure	tonic-clonic with fever
Frequency of initial sz	2/d-1/3 m	7–8/y	NA	1 /m	NA
Transition to other sz	tonic-clonic with/without fever	no	tonic-clonic with fever, focal seizure	tonic-clonic	no
Initial EEG, age	normal	multifocal sharp wave	NA	NA	NA
EEG follow up	multifocal sharp wave spike wave, sharp and slow wave complex and spike and slow wave complex(11 m)	occipital spike wave and spike and slow wave complex(7 y)	spike wave and spike and slow wave complex	occipital spike wave and spike and slow wave complex(12 y)	NA
MRI findings	delayed myelination	normal	NA	normal	NA
drugs	VPA+LEV	VPA+LEV+TPM	VPA+TPM, OXC, CZP	TPM, CBZ	no
Sz prognosis	no effect	seizure free	seizure reduction	no effect	seizure free
Development	normal(15 m)	normal(7 y)	severe developmental delay(7 y)	NA	normal(5 y)

Patient	26	27	28	29	30

Sex	F	F	F	M	F
history of pregnancy and delivery	normal	NA	normal	normal	normal
Family history	mother	NA	(-)	Grand-uncle	(-)
Diagnosis	GEFS+	Dravet syn	epilepsy	GEFS+	Dravet syn
Mutation	NM_001165963:exon21:c.4009 G>A(p.V1337M),NA	NM_001165963.1:exon14:c.2589+3 A>T, *de novo*	NM_001165963:exon11:c.1738C>T(p.R580X),*de novo*	NM_001165963:exon2:c.301 C>T(p.R101W),NA	NM_001165963:exon11:c.2021 A>G(p.D674G), NA
HGMD	N	Y	Y	Y	Y
Age at onset	18 m	6 m	2 m	3 m	6 m
Initial epileptic attacks	tonic-clonic with fever	tonic-clonic	tonic without fever	tonic-clonic with fever	focal seizure
Frequency of initial sz	2/3 y	NA	2 /m	1 /m	NA
Transition to other sz	NA	NA	tonic-clonic, focal seizures,SE	no	no
Initial EEG, age	NA	normal	normal	normal	normal
EEG follow up	NA	normal	spike wave and spike and slow wave complex(6 m)	multifocal spike wave and spike and slow wave complex	normal
MRI findings	NA	NA	normal	normal	normal
drugs	no	VPA+LEV	LEV+TPM, OXC	VPA+LEV	VPA+TPM+CZP, LEV
Sz prognosis	seizure free	seizure free	seizure reduction	no effect	seizure reduction
Development	NA	mild developmental delay(9 y)	normal(1 y)	normal(2 y)	severe developmental delay(3 y)

Patient	31	32	33

Sex	M	M	M
history of pregnancy and delivery	normal	NA	normal
Family history	father	NA	(-)
Diagnosis	GEFS+	epilepsy	Dravet syn
Mutation	NM_001165963:exon26:c.5293 T>A(p.F1765I), paternal	NM_001165963:exon25:c.4633 A>G(p.I1545V), *de novo*	NM_001165963:exon21:c.4143delT(p.G1382Vfs⁎9),NA
HGMD	N	Y	N
Age at onset	14 m	3 m	6 m
Initial epileptic attacks	tonic	focal seizure	tonic
Frequency of initial sz	NA	NA	1–2 /m
Transition to other sz	focal seizures	tonic-clonic	focal seizures, SE
Initial EEG	abnormal	abnormal	multifocal sharp wave and sharp and slow wave complex
EEG follow up	normal	abnormal	multifocal sharp wave, spike wave, sharp and slow wave complexand spike and slow wave complex(15 m)
MRI findings	arachnoid cyst	normal	normal
drugs	VPA	VPA+LEV, OXC, ketogenic diet	VPA, ketogenic diet
Sz prognosis	seizure free	no effect	seizure reduction
Development	NA	normal(6 y)	mild developmental delay(18 m)

Dravet syn: Dravet syndrome, FS+: febrile seizures plus, GEFS+: Generalized epilepsy with febrile seizures plus, VPA: valproate, LEV: levetiracetam, TPM: Topamax, CBZ: carbamazepine, CZP: clonazepam, OXC: oxcarbazepine; sz: seizure(s), SE: status epilepticus.

**Table 2:** Summary of the clinical features of subjects with a *SCN2A* mutation.

Patient	34	35	36	37	38
Sex	M	M	F	M	F
history of pregnancy and delivery	normal	asphyxia	normal	normal	normal
Family history	(-)	(-)	(-)	uncle	(-)
Diagnosis	neonatal seizure	neonatal seizure	epileptic encephalopathy	epilepsy	epilepsy
Mutation	NM_021007:exon26:c.4751 C>G(p.S1584C),NA	NM_021007:exon27:c.4918 A>T(p.I1640F),NA	NM_021007:exon7:c.781 G>A(p.V261M), *de novo*	NM_021007:exon17:c.2989 G>T(p.D997Y), *de novo*	NM_021007:exon27:c.4886 G>A(p.R1629H),NA
HGMD	N	N	Y	N	N
Age at onset	3d	7d	4d	1d	1d
Initial epileptic attacks	clonic	clonic	tonic-clonic	clonic	tonic
Frequency of initial sz	NA	frequently	frequently	6–7/d	1/d
Transition to other sz	NA	NA	no	tonic-clonic	tonic-clonic
Initial EEG, age	left frontal-parietal sharp wave,spike wave and sharp and slow wave complex	slow wave	multifocal spikeand slow wave complex and sharp and slow wave complex	suppression-burst	slow wave
EEG follow up	NA	NA	NA	NA	NA
MRI findings	normal	bilateral parietal,occipital and basal ganglia region injury, myelination delayed	normal	normal	cystic change of the body of lateral ventricle
drugs	phenobarbital	phenobarbital, VPA+LEV	phenobarbital, LEV	phenobarbital, LEV+TPM	VPA+LEV
Sz prognosis	seizure free	no effect	seizure free	seizure free	seizure free
Development	NA	NA	severe developmental delay	severe developmental delay	normal

Patient	39	40	41	42	43
Sex	F	M	M	M	M
history of pregnancy and delivery	normal	normal	normal	normal	normal
Family history	(-)	(-)	(-)	(-)	(-)
Diagnosis	neonatal seizure	neonatal seizure	epileptic encephalopathy	epileptic encephalopathy	epilepsy
Mutation	NM_021007:exon2:c.106 A>G(p.R36G),NA	NM_021007:exon16:c.2670 C>G(p.I890M),NA	NM_021007:exon15:c.2558 G>A(p.R853Q), *de novo*	NM_021007:exon27:c.4886 G>A(p.R1629H), *de novo*	NM_021007:exon21:c.3961 G>A(p.E1321K), *de novo*
HGMD	N	N	Y	N	Y
Age at onset	2d	1d	7 m	1 m	3d
Initial epileptic attacks	clonic	focal seizures	eyelid myoclonus	focal seizures	eyelid myoclonus, tonic-clonic
Frequency of initial sz	3–5/d	5–8/d	NA	NA	7–8/d
Transition to other sz	NA	NA	spasms, tonic-clonic	spasms	NA
Initial EEG, age	multifocal sharp wave and sharp and slow wave complex	multifocal spikeand slow wave complex and sharp and slow wave complex	multifocal spikeand slow wave complex and sharp and slow wave complex	multifocal spikeand slow wave complex and sharp and slow wave complex	multifocal spikeand slow wave complex and sharp and slow wave complex
EEG follow up	NA	NA	NA	NA	normal
MRI findings	normal	normal	cerebral dysplasia and thin corpus callosum	thin corpus callosum	thin corpus callosum
drugs	NA	NA	CBZ+VPA+ mexiletine	VPA+LEV, OXC, CZP	VPA+LEV+TMP
Sz prognosis	seizure free	seizure free	no effect	no effect	seizure free
Development	NA	NA	severe developmental delay	severe developmental delay	normal

Patient	44	45	46	47	48

Sex	M	M	F	F	M
history of pregnancy and delivery	normal	normal	normal	normal	NA
Family history	(-)	mother, elder sister	(-)	(-)	NA
Diagnosis	epileptic encephalopathy	BFIS	epilepsy	epileptic encephalopathy	epilepsy
Mutation	NM_021007:exon27:c.5616 G>A(p.M1872I), *de novo*	NM_021007:exon2:c.209 C>T(p.P70L),NA	NM_021007:exon19:c.3524 G>A(p.C1175Y),NA	NM_021007:c.3521–2 A>G, *de novo*	NM_021007:exon27:c.5045 T>C(p.F1682S), *de novo*
HGMD	N	N	N	N	N
Age at onset	3 m	1 y	4 y	19 m	7 y
Initial epileptic attacks	tonic	atonic seizure	tonic	tonic-clonic	NA
Frequency of initial sz	7/10d	4/d	NA	NA	NA
Transition to other sz	NA	NA	atonic seizures	NA	NA
Initial EEG	NA	abnormal	multifocal spikeand slow wave complex and sharp and slow wave complex	epileptiform discharge	NA
EEG follow up	normal	normal	NA	normal	NA
MRI findings	normal	NA	normal	normal	NA
drugs	VPA, LEV	no	VPA	VPA+TPM	CBZ
Sz prognosis	seizure free	seizure free	seizure free	seizure reduction	seizure free
Development	developmental delay	normal	severe developmental delay	developmental delay	developmental delay

VPA: valproate, LEV: levetiracetam, TPM: Topamax, CBZ: carbamazepine, CZP: clonazepam, OXC: oxcarbazepine, sz: seizure(s), SE: status epilepticus.

## 2 Experimental design, materials and methods

Eight hundred and twenty-two children with epilepsy or neonatal severe seizures were performed whole exome sequencing (WES) or clinical exome sequencing (focused 2742 genes). We extracted genomic DNA in the peripheral blood of the children. DNA fragments were enriched for whole exome sequencing using the Agilent (Santa Clara, CA, USA) SureSelectXT Human All Exon 50 Mb kit, and for panel sequencing using the Agilent (Santa Clara, CA, USA) ClearSeq Inherited Disease panel kit. Our own database and public databases including the exome aggregation consortium, the 1000 Genome Project, and the dbSNP137 reported in the UCSC Genome Browser were used to screen the variants. Pathogenic variants or likely pathogenic variants were considered to be candidate variants. The candidate variants were validated by Sanger sequencing. *SCN1A* or *SCN2A* mutations were recruited in the database. Clinical information including sex, birth history, family history, seizure onset, seizure types, frequency of seizures, initial and follow-up EEGs, brain MRI findings, antiepileptic drugs, prognosis and developmental data were collected.
